# Determinants of Parents' and Adolescents' Motivation Before Pediatric Weight Management—Data From the STARKIDS Cohort

**DOI:** 10.1002/oby.70185

**Published:** 2026-04-12

**Authors:** Anne Herschbach, Rebecca Erschens, Florian Junne, Stefan Ehehalt, Johann Jacoby, Peter Martus, Isabelle Mack, Alisa Weiland, Inga Krauß, Constanze Greule, Guido Zurstiege, Annalena Wels, Sophia Helen Adam, Andreas Hoell, Wolfgang Bethge, Monika Ums, Torben Sammet, Maren Reyer, Olaf Schliesing, Stephan Zipfel, Caterina Gawrilow, Katrin Elisabeth Giel, Katrin Ziser

**Affiliations:** ^1^ Department of Psychosomatic Medicine and Psychotherapy University Hospital Tuebingen Tuebingen Baden‐Wurttemberg Germany; ^2^ Department of Psychosomatic Medicine and Psychotherapy Otto von Guericke University Magdeburg Magdeburg Saxony‐Anhalt Germany; ^3^ Public Health Department of Stuttgart Stuttgart Baden‐Wurttemberg Germany; ^4^ Institute of Clinical Epidemiology and Applied Biometry, Medical Faculty University of Tuebingen Tuebingen Baden‐Wurttemberg Germany; ^5^ Department of Sports Medicine Medical University Hospital Tuebingen Tuebingen Baden‐Wurttemberg Germany; ^6^ Interfaculty Research Institute for Sports and Physical Activity University of Tuebingen Tuebingen Baden‐Wurttemberg Germany; ^7^ Institute of Sports Science University of Tuebingen Tuebingen Baden‐Wurttemberg Germany; ^8^ Insitute of Media Studies University of Tuebingen Tuebingen Baden‐Wurttemberg Germany; ^9^ Clinic for Psychiatry and Psychotherapy, Central Institute of Mental Health Medical Faculty Mannheim, University of Heidelberg Mannheim Baden‐Wurttemberg Germany; ^10^ Center for Clinical Trials, Medical Faculty University of Tuebingen Tuebingen Baden‐Wurttemberg Germany; ^11^ Baden‐Wuerttemberg State Health Office Stuttgart Baden‐Wurttemberg Germany; ^12^ Data Medico GmbH Heidelberg Baden‐Wurttemberg Germany; ^13^ German Centre for Mental Health (DZPG), partner site Tuebingen Tuebingen Baden‐Wurttemberg Germany; ^14^ Department of Psychology University of Tuebingen Tuebingen Baden‐Wurttemberg Germany

## Abstract

**Objective:**

Pediatric obesity (OB) treatments show small effects and high dropout rates, potentially due to low motivation in participating families. Motivation prior to a weight management program is referred to as “readiness to change” (RTC). Understanding RTC is key to improving outcomes. Previous studies observed determinants for RTC, but results are heterogenous and data about adolescents' RTC are lacking.

**Methods:**

Baseline data of the STARKIDS trial were used to find determinants of RTC in (a) adolescents (*n* = 125), (b) their caretakers (*n* = 185), and (c) caretakers of younger children (*n* = 325). Potential determinants were sociodemographic variables, weight, general quality of life (QoL), OB‐related QoL, self‐efficacy, and body image of caretakers and children. Pearson correlations (*t*‐tests and ANOVA) and multiple linear regression analyses were performed.

**Results:**

The RTC of adolescents and of both groups of caretakers was determined by adolescents'/children's OB‐related QoL. The RTC of caretakers of younger children was also determined by children's BMI‐SDS_LMS_.

**Conclusions:**

OB‐related QoL plays a major role in determining adolescents' and caretakers' motivation prior to weight management. In all groups, most significant determinants refer to corresponding family members, showing that the mutual influence is large.

**Trial Registration:**

German Clinical Trials Register (DRKS) DRKS000228 13 (acknowledged primary register of the World Health Organization).

## Study Importance

- What is already known?
  ○ Knowledge on motivation before a weight management program for children and adolescents suffering from obesity and overweight is pivotal because it can impact the outcomes.
  ○ Readiness to change, as one aspect of motivation, means a “willingness” to change specific behaviors and is of interest before families begin participation in a weight management program.
  ○ Different determinants of parental readiness to change have been observed (e.g., parental weight, children's sex), but research in adolescents is lacking.
- What does this study add?
  ○ Obesity‐related quality of life determines adolescents' own motivation and the motivation of caretakers of children and adolescents.
  ○ The readiness to change of caretakers of younger children (aged 3–11 years) is additionally determined by the children's BMI‐SDS_LMS_.
- How might these results change the direction of research or the focus of clinical practice?
  ○ Families seem to be more motivated to participate in a pediatric weight management program when children and adolescents are experiencing a lower obesity‐related quality of life.
  ○ Clinicians could use obesity‐related quality of life to boost motivation by educating about the serious health risks of obesity and overweight, as well as the benefits of weight management, such as improved body image and easier movement.

## 1 Introduction

The prevalence of obesity (OB) in children and adolescents has tripled since 1990 [1]. In 2022, the worldwide prevalence of OB in 5‐year‐olds was 11.7% in boys and 8.4% in girls. In 19‐year‐olds the prevalence was 5.7% in male and 6.2% in female adolescents in 2022 [2]. Consequences thereof are somatic, psychological, and psychosocial comorbidities [3, 4], as well as a high financial burden on the health care system [5]. Treatment of OB and overweight (OW) in children and adolescents should therefore start as early as possible. Guideline‐based treatment is participation in a multidisciplinary weight management program, consisting of nutrition, structured physical activity, counseling, and specialist support for behavior changes, which involve the whole family [6, 7, 8]. Systematic reviews show that affected children and adolescents can benefit from such weight management programs [9], but effects are often small [9] and dropout rates are high [10]. Reasons for this include low motivation among participating families [11].

To reduce dropout rates, avoid frustration, and ultimately save treatment costs, knowledge on treatment motivation in pediatric OB is pivotal. Although motivation seems to play a major role in childhood weight management and treatment guidelines recommend fostering treatment motivation [6, 7], not all treatment programs actively address motivation. Evidence suggests that motivation can be enhanced during a weight reduction program (e.g., [12, 13]). Some studies have also found that motivation is related to treatment outcomes or dropout (e.g., [14, 15]), but its predictive value for weight loss remains unclear [16, 17]. However, due to a heterogeneity in the measurement of motivation, generalized conclusions can hardly be drawn [18].

One aspect of motivation, especially important in upcoming behavior changes, is the construct of readiness to change (RTC). Readiness means “willingness” or being open to specific behavior changes [19]. The construct is based on the Transtheoretical Model (TTM), a theory explaining behavior change. According to TTM, a change toward a healthier behavior takes place over different stages, the stages of change [20, 21]. Motivation is needed throughout this whole process. The RTC score is calculated from these stages of change [19, 22]. Ceccarini et al. [23] posit that RTC is among the most important factors influencing behavior change, such as weight management.

Different authors found several determinants of RTC in families prior to participation in weight management: Most studies assessed determinants for parental motivation or RTC regarding treatment for their children's OB/OW. Ramel et al. [24] found higher motivation in parents with lower educational level and higher income and in parents considering themselves as Black or non‐Hispanic. Maximova et al. [25] observed that parents with higher motivation were less OW and had a more healthy lifestyle compared to parents with lower motivation. Taylor et al. [26] investigated the association between children's variables and parental motivation: They observed that parents showed greater motivation when their children were girls, had higher weight, or were less physically active. Furthermore, parents were more motivated when they had concerns about their children's weight. Finally, Rhee et al. [27] observed that parents showed higher motivational levels when their children were older, when they perceived themselves to be affected by OW, and when they thought that their child's weight was a problem. Only one study investigated determinants for children's own motivation: Ramel et al. [24] observed that higher household income and higher parental education were associated with lower readiness in children. Children in this study were aged up to 12. To the best of our knowledge, there is no study which investigated determinants of motivation in adolescents (aged 12 and above).

Another concept, closely linked to motivation, is self‐efficacy, as it is also part of TTM [28]. Self‐efficacy refers to a person's confidence in his/her ability to successfully complete the challenges he/she is facing [29]. It is related to performance and success in weight management programs [30, 31, 32] wherefore it is trying to be addressed in these and wherefore it might also be related to motivation prior to weight management. Next to that, body image is another variable that plays a role in weight management and might be related to families' motivation. Body image means a mental representation a person has of his or her own body [33]. Previous studies observed that pretreatment body image in adults is associated with weight loss [34]. In children, it can be increased through participation in weight management programs [35]. Lastly, quality of life (QoL) is another variable of interest since it is one reason for adolescents to participate in weight management [36]. In adults there are findings that higher motivation is associated with higher QoL [37]. Moreover, there is a specific QoL, namely OB‐related QoL, which is of importance here because it focuses on the specific burden faced when suffering from OB/OW. Therefore, general QoL and OB‐related QoL are important concepts in the field of motivation prior to pediatric weight management.

Thus, currently there is a wide range of already reported and further possible determinants for parental motivation to participate in a childhood weight management program. Possible reasons for the heterogeneity of determinants could be different measures and operationalizations of motivation [18]. Additionally, it is striking that most studies only examined factors that influence parental readiness. Adolescent's own readiness is barely examined [24]. Especially in adolescents who become increasingly independent [38], it is crucial to examine their own motivation and to identify similarities and differences between them and their parents.

In order to address this research gap, we report baseline data from the large‐scale intervention trial STARKIDS, which investigated an e‐health supported family‐based weight management program for pediatric OB and OW [39]. STARKIDS is an acronym for the German title “Stufenmodell Adipositaspraevention und ‐therapie im Kindes‐ und Jugendalter” (English: stepwise model for obesity prevention and treatment in children and adolescents). We separately examined determinants of motivation of (a) affected adolescents (aged 12 and older), (b) their parents and caretakers, and (c) parents/caretakers of younger children (aged 11 and younger) before participation in the STARKIDS trial. We had the following research question: Which factors determine adolescents' (aged 12 and older) own motivation before beginning a weight management program, as well as their caretakers' motivation and the motivation of caretakers of younger children (aged 11 and younger)? As potential determinants we included variables of previous research (age, sex, level of education, income, migration background, and weight) and further variables of interest (QoL, OB‐related QoL, self‐efficacy, and body image) of caretakers and adolescents.

## 2 Methods

### 2.1 Study Design

This study is a secondary analysis based on a cross‐sectional analysis of the baseline data of the STARKIDS study [39]. The primary aim of STARKIDS was to develop and evaluate a weight management program for families with children or adolescents affected by OB and OW [39]. Baseline data were assessed between April 2022 and June 2023. The STARKIDS study was preregistered in the German Clinical Trials Register (DRKS) DRKS000228 13 (acknowledged primary register of the World Health Organization) on November 27, 2020 (Universal Trial Number U1111‐1254‐9536). Ethical approval of this study was obtained from the ethics committee of the Medical Faculty of the University of Tuebingen (no. 620/2020BO1), as well as from the ethics committee of the medical association of the state of Baden‐Wuerttemberg (no. B‐F‐2020‐160).

### 2.2 Participants

Participants were children and adolescents with OB or OW and one of their caretakers (mostly parents). Recruitment of the families was done by participating pediatric and family practices in the federal state of Baden‐Wuerttemberg in southern Germany. Inclusion criteria for the children and adolescents were age between 3 and 17 years, BMI > 90th percentile [40, 41], being in statutory health insurance, and adequate knowledge of German. Exclusion criteria were a prior participation in an interdisciplinary weight management program for at least 1 year, inpatient treatment of OW/OB within the last year, other severe systemic diseases, and severe psychosocial stress or diseases which were not associated with OB/OW. For the current analysis, three samples were taken from the STARKIDS cohort: (a) adolescents aged 12 and older, (b) caretakers of adolescents aged 12 and older (87.1% mothers), and (c) caretakers of children aged 11 and younger (85.9% mothers). A total of 611 dyads of caretakers and children/adolescents were recruited to the STARKIDS study, but not all attended baseline measures. Therefore, there are no data available on these families. Baseline measures were assessed from *n* (sample a) = 199, *n* (sample b) = 199, and *n* (sample c) = 343. Participants were excluded if the outcome variable was missing, resulting in the following sample sizes for the current analysis: *n* (sample a) = 125, *n* (sample b) = 185, and *n* (sample c) = 325.

### 2.3 Procedure

After recruitment and eligibility checking in the pediatric or family practice, a physical examination of the child/adolescent was conducted by the family doctor, where anthropometric data were assessed. In addition, families completed a web‐based questionnaire at home. For each child‐caretaker dyad, the caretaker filled out the questionnaire with sociodemographic data and standardized questionnaires. Adolescents aged 12 and above had the opportunity to additionally complete a web‐based questionnaire on their own. Since this questionnaire was voluntary, the group of caretakers was larger than the group of adolescents themselves.

### 2.4 Measurements

Detailed information about included questionnaires is listed in Table 1. Motivation was operationalized as RTC according to DiClemente et al. [19] and Carbonari et al. [22] and assessed as part of the questionnaire. The RTC score is calculated from the subscales of the University of Rhode Island Change Assessment (URICA) [42, 43, 54]. This questionnaire is based on TTM as most motivational measures are based on that [23]. The RTC score is calculated by summing up the means of the subscales Contemplation, Action, and Maintenance and then subtracting the mean of the subscale Precontemplation [19].

**TABLE 1:** Description of the questionnaires used for the current analysis.

Questionnaire	Construct	Subscales, ranges, used score, interpretation	Example item^a^	Reliability and validity	Completer (about)
University of Rhode Island Change Assessment‐Short (URICA‐S) [42]	Stages of change according to TTM, RTC score	Four subscales: precontemplation, contemplation, action, maintenance; range 0–4; RTC score used (range −4 to 12), higher values indicating higher RTC	I'm not the problem one. It doesn't make much sense for me to be here (precontemplation); I wish I had more ideas on how to solve the problem (contemplation); I am really working hard to change (action); I'm here to prevent myself from having a relapse of my problem (maintenance),	Reliability: Cronbach's *α* = 0.61–0.84	Adolescent (self‐report)
Parent Perspective University of Rhode Island Change Assessment‐Short (PURICA‐S) [43]	Parental stages of change (related to their children's problem) according to TTM, RTC score	Four subscales: precontemplation, contemplation, action, maintenance; range 0–4; RTC score used (range −4 to 12), higher values indicating higher RTC	My child does not have a problem; therefore, it does not make sense for us to be here (precontemplation); I wish I had more ideas about how to solve my child's problem (contemplation); I am working hard to bring about change for my child (action); I am here to prevent my child from relapsing (maintenance).	Reliability: Cronbach's *α* = 0.30–0.84	Parent (self‐report)
Eating Disorder Inventory‐2 (EDI‐2) [44, 45, 46]	Body image, body dissatisfaction	Only subscale body dissatisfaction used; range 1–6; sum used, higher values indicating higher body dissatisfaction	I think that my stomach is too big.	Reliability: *r*_tt_ = 0.89/0.94 Validity: *r* = 0.35 with Beck Depression Inventory	Parent (self‐report)
Eating behavior inventory‐Child (IEG‐K) [47]	Body image	Only subscale body image used; range 0–3; mean*10 used, higher values indicating higher body dissatisfaction	I am satisfied with my figure.	Reliability: Cronbach's *α* = 0.88	Adolescent (self‐report)
Self‐efficacy scale [48, 49]	General self‐efficacy	One scale; range 1–4; mean used, higher values indicating higher self‐efficacy	I can solve most problems if I invest the necessary effort.	Reliability: Cronbach's *α* = 0.78–0.79	Adolescent (self‐report)
Self‐efficacy in education [50]	Parents‘ expectation regarding their abilities to educate their children	One scale; range 0–3; mean used, higher values indicating higher self‐efficacy	It is easy for me to be a loving mother/father.	Reliability: Cronbach's *α* = 0.78/0.79 Validity: *r* = 0.63/0.64 with parent behavior in risk situation	Parent (self‐report)
Revidierter Fragebogen für Kinder und Jugendliche zur Erfassung der gesundheits‐bezogenen Lebensqualität^b^ (KINDL‐R), version for parents (different versions for parents of children aged 3–6 and aged 7–17), including OB module [51, 52, 53]	Quality of life, OB‐related quality of life	Six subscales: body, mind, self‐esteem, family, friends, school; In addition: OB‐related quality of life; range 1–5; sum score of 100 used, higher values indicating higher quality of life; one score for general quality of life (consisting of the subscales) and one score for OB‐related quality of life	During the past week my child was proud of himself (self‐esteem); During the past week my child got out of breath quickly and was puffed out quickly (OB‐related quality of life).	Reliability: Cronbach's *α* = 0.89	Parent (proxy report about child/adolescent)
Revidierter Fragebogen für Kinder und Jugendliche zur Erfassung der gesundheits‐bezogenen Lebensqualität^b^ (KINDL‐R), version for adolescents (different versions for age groups 12–13 and 14–17), including OB module [51, 52, 53]	Quality of life, OB‐related quality of life	See above	During the past week I was proud of myself (self‐esteem); During the past week I got out of breath quickly and I was puffed out quickly (OB‐related quality of life).	Reliability: Cronbach's *α* = 0.84	Adolescent (self‐report)

^a^ Item text is translated to English from the German version which was used.

^b^ In English: Revised questionnaire for children and adolescents to assess health‐related quality of life.

The following other variables were assessed: Children's and adolescents' age, sex, and BMI‐SDS_LMS_ were assessed at the physical examination in the pediatric or family practice. Parental BMI was self‐reported in the web‐based questionnaire. Sociodemographic data (migration background, family income, and caretakers' education) were part of the caretakers' questionnaire. Migration background of caretakers and minors was operationalized based on Schenk et al. [55]: Caretakers were considered as having a migration background if they reported a different nationality than German or having a history of migration. Children and adolescents were considered as having a migration background if both parents had one or if their birth year was before the mother's migration. Income was operationalized as monthly household net income, and caretaker's education was operationalized as the level of their school leaving certificate. Further variables were QoL and OB‐related QoL, both assessed with the KINDL‐R questionnaire, [51, 52, 53], self‐efficacy, assessed with the self‐efficacy scale and the self‐efficacy in education scale [48, 49, 50], and body image, assessed with the Eating Disorder Inventory‐2 and the Eating behavior inventory‐Child [44, 45, 46, 47]. QoL and OB‐related QoL were self‐reported in adolescents. Parents had to proxy‐report their children's and adolescents' QoL. Body image and self‐efficacy were assessed as self‐reports in caretakers and adolescents. All these measurements were part of the web‐based questionnaire separately for adolescents and caretakers. Table 1 summarizes all details.

### 2.5 Statistical Analysis

IBM SPSS Statistics for Windows, Version 28.0.0.0 (190), was used for data analyses. Sample characteristics are described in means and standard deviations (SD) or percentages. Table 2 provides an overview of variables used as possible determinants in the three samples. First, Pearson correlations (*t*‐tests and one‐way ANOVA for variables not measured at interval level) were calculated to minimize the pool of possible predictors. Factors for which significant correlations were found were included in the multivariate regression analyses. A multivariate regression analysis was conducted for each sample. Outcomes were RTC of (a) adolescents, (b) caretakers of adolescents, and (c) caretakers of children. In all regression models, all variables were entered simultaneously (forced entry). The following assumptions for multiple linear regression were tested: linearity, homoscedasticity, no outliers, no multicollinearity, independent and normally distributed residuals. No violations were observed in all models. The level of statistical significance was defined as *p* < 0.05.

**TABLE 2:** Variables included as possible determinants in the three samples.

Sample a: Adolescents' RTC	Sample b: RTC of caretakers from adolescents (12 and older)	Sample c: RTC of caretakers from children (11 and younger)
BMI‐SDS_LMS_	Adolescents' BMI‐SDS_LMS_	Children's BMI‐SDS_LMS_
	Own BMI	Own BMI
Age	Adolescents' age	Children's age
Sex	Adolescents' sex	Children's sex
Household income	Household income	Household income
Migration background	Migration background	Migration background
	Education level	Education level
Body image	Own body image	Own body image
Self‐efficacy	Self‐efficacy in parenting	Self‐efficacy in parenting
Quality of life	Quality of life of adolescent (proxy report)	Quality of life of children (proxy report)
OB‐related quality of life	OB‐related quality of life of adolescent (proxy report)	OB‐related quality of life of children (proxy report)
Caretakers' RTC		

## 3 Results

Demographic data, as well as descriptive questionnaire data, are presented in Table 3. Most children and adolescents (82.84%) suffered from OB (i.e., BMI > 97th percentile).

**TABLE 3:** Participant demographics and descriptive data of possible determinants.

Variable	Sample a: Adolescents (*n* = 125)	Sample b: Caretakers of adolescents (*n* = 185)	Sample c: Caretakers of children (*n* = 325)
	Mean (SD) or frequency in %	Mean (SD) or frequency in %	Mean (SD) or frequency in %
Age	14.13 (1.45)	43.02 (6.07)	39.18 (6.70)
Age of associated child/adolescent (if applicable)		13.89 (1.39)	8.97 (2.32)
Sex, female	40.0%	88.0%	88.6%
Sex, female of associated child/adolescent (if applicable)		39.5%	46.8%
BMI	BMI‐SDS_LMS_: 2.39 (0.47); BMI percentile: 98.52 (1.66)	29.76 (6.49)	29.80 (6.39)
BMI of associated child/adolescent (if applicable)		BMI‐SDS_LMS_: 2.38 (0.48); BMI percentile: 98.47 (1.67)	BMI‐SDS_LMS_: 2.39 (0.52); BMI percentile: 98.41 (1.78)
Family income			
< 1000 €	2.4%	3.8%	5.2%
1000–1999 €	17.6%	20.5%	15.7%
2000–2999 €	32.0%	27.6%	27.7%
3000–3999 €	23.2%	24.3%	23.1%
4000–4999	13.6%	16.8%	17.8%
> 5000 €	6.4%	6.5%	9.2%
Migration background	32.0%	47.6%	48.0%
Level of school education^a^			
Lower secondary		25.9%	24.0%
Intermediate secondary		38.9%	40.3%
A‐levels		23.2%	28.0%
None		3.2%	3.7%
Special education		0.5%	0.3%
Other		7.0%	2.8%
Body image^b^	20.83 (6.47) (self‐report)	35.61 (12.15) (self‐report)	37.60 (11.40) (self‐report)
Self‐efficacy^b^	2.67 (0.53) (self‐report)	2.09 (0.42) (self‐report)	2.13 (0.40) (self‐report)
Quality of life of adolescents/children	65.02 (13.86) (self‐report)	68.00 (11.51) (proxy report)	74.77 (11.09) (proxy report)
OB‐related quality of life of adolescents/children	63.36 (19.16) (self‐report)	67.07 (17.19) (proxy report)	73.08 (17.08) (proxy report)
URICA‐S/PURICA‐S scales			
Precontemplation	0.87 (0.70)	0.53 (0.60)	0.48 (0.53)
Contemplation	2.46 (1.00)	2.69 (0.93)	2.68 (0.97)
Action	2.15 (0.84)	2.40 (0.88)	2.48 (0.90)
Maintenance	1.56 (1.09)	1.52 (1.04)	1.37 (1.08)
RTC score	5.29 (2.65)	6.09 (2.48)	6.06 (2.57)

^a^ The German terms for the different school levels are: Hauptschulabschluss, Realschulabschluss, Abitur. Pupils are allocated according to their school performances.

^b^ Body image and self‐efficacy were assessed with different questionnaires for adolescents and parents (see Table 1).

### 3.1 Determinants of Adolescents' RTC

The mean level of RTC in the sample of adolescents was 5.31 (SD = 2.68). Table 4 shows the results of the Pearson correlation analyses. There were significant correlations between adolescents' RTC and their body image, their OB‐related QoL, and their caretakers' RTC (Table 4). Therefore, only these variables were included in the multiple linear regression (Table 5). The model was able to significantly determine adolescents' RTC, *F*(3, 113) = 8.86, *p* < 0.001. The *R* ^2^ for the overall model was 0.19 (adjusted *R* ^2^ = 0.17). Looking at the statistical significance of the individual coefficients, only the variable of OB‐related QoL (*β* = −0.31, *p* = 0.002) could significantly determine adolescents' RTC. Caretakers' RTC showed a clear trend but did not reach significance (*β* = 0.17, *p* = 0.053). The variable body image did not reach significance and could therefore not determine adolescents' RTC.

**TABLE 4:** Pearson correlations, *t*‐tests, and one‐way ANOVA for adolescents' and caretakers' RTC and possible determinants.

Pearson correlations	Adolescents' RTC		Caretakers' (of adolescents aged 12 and older) RTC		Caretakers' (of children aged 11 and younger) RTC	
	*r*	*p*	*r*	*p*	*r*	*p*
BMI‐SDS_LMS_ of child/adolescent	0.09 (*N* = 125)	0.298	0.11 (*N* = 185)	0.127	0.19 (*N* = 325)	< 0.001***^c^
BMI of caretaker			0.02 (*N* = 177)	0.777	0.11 (*N* = 303)	0.064
Age of child/adolescent	0.09 (*N* = 125)	0.312	0.07 (*N* = 185)	0.356	0.05 (*N* = 325)	0.334
Family income	0.03 (*N* = 119)	0.715	−0.03 (*N* = 184)	0.741	0.02 (*N* = 321)	0.690
Body image of adolescent	0.28 (*N* = 125)	0.002**	n/a	n/a	n/a	n/a
Body image of caretaker	n/a	n/a	0.03 (*N* = 181)	0.711	0.12 (*N* = 322)	0.028*
Self‐efficacy of adolescent	−0.06 (*N* = 125)	0.512	n/a	n/a	n/a	n/a
Self‐efficacy in education of caretaker	n/a	n/a	−0.09 (*N* = 182)	0.218	−0.14 (*N* = 322)	0.012*
Quality of life^a^	−0.15 (*N* = 124)	0.105	−0.26 (*N* = 182)	< 0.001***	−0.20 (*N* = 319)	< 0.001***
OB‐related quality of life^a^	−0.38 (*N* = 124)	< 0.001***	−0.44 (*N* = 184)	< 0.001***	−0.30 (*N* = 322)	< 0.001***
RTC of caretaker	0.28 (*N* = 118)	0.002**	n/a	n/a	n/a	n/a

*t*‐tests	Mean (SD)	*t*	*p*	Mean (SD)	*t*	*p*	Mean (SD)	*t*	*p*
Sex of child/adolescent	(*N* = 125)	0.89	0.376	(*N* = 185)	−1.31	0.192	(*N* = 325)	−0.51	0.614
Male	5.46 (2.60)			5.89 (2.61)			5.99 (2.68)		
Female	5.03 (2.72)			6.38 (2.24)			6.13 (2.46)		
Migration background of child/adolescent	(*N* = 125)	0.31	0.759	n/a	n/a		n/a	n/a	
Yes	5.18 (2.91)								
No	5.34 (2.53)								
Migration background of caretakers	n/a	n/a		(*N* = 185)	−0.36	0.722	(*N* = 325)	0.16	0.871
Yes				6.15 (2.50)			6.03 (2.52)		
No				6.02 (2.47)			6.08 (2.63)		

ANOVA	Mean (SD)	*F*	*p*	Mean (SD)	*F*	*p*	Mean (SD)	*F*	*p*
Level of education of caretaker^b^	n/a	n/a		(*N* = 163)	0.25	0.781	(*N* = 300)	1.03	0.357
Lower secondary				5.99 (2.69)			5.77 (2.76)		
Intermediate secondary				6.28 (2.41)			6.03 (2.64)		
A‐levels				6.29 (2.01)			6.34 (2.17)		

^a^ Quality of life and OB‐related quality of life are self‐reported in adolescents and proxy‐reported in caretakers.

^b^ Small categories of the variable education were let out to calculate ANOVA.

^c^ Significance codes: *p < 0.05; **p < 0.01; ***p < 0.001.

**TABLE 5:** Results of the multiple regression analyses for the three samples.

	*B*	SE	95% CI	*β*	*p*
Sample a: Adolescents’ RTC (*n* = 117)					
Constant	6.21	1.67	[2.91, 9.52]	—	< 0.001***^a^
Body image of adolescent	0.04	0.04	[−0.04, 0.12]	0.09	0.364
OB‐related quality of life (self‐report adolescent)	−0.04	0.01	[−0.07, −0.02]	−0.31	00.002**
RTC of caretaker	0.17	0.09	[−0.00, 0.35]	0.17	0.053
*R* ^*2*^ = 0.19, adj. *R* ^*2*^ = 17					
Sample b: Caretakers’ (of adolescents) RTC (*n* = 181)					
Constant	10.34	0.99	[8.39, 12.28]	—	< 0.001***
Quality of life (proxy‐report of parent)	−0.00	0.02	[−0.04, 03]	−0.01	0.921
OB‐related quality of life (proxy‐report of parent)	−0.06	0.01	[−0.08, −0.04]	−0.44	< 0.001***
*R* ^*2*^ = 0.19, adj. *R* ^*2*^ = 0.18					
Sample c: Caretakers’ (of children) RTC (*n* = 315)					
Constant	7.82	1.40	[5.07, 10.57]	—	< 0.001***
BMI‐SDS_LMS_ of child/adolescent	0.75	0.27	[0.23, 1.27]	0.16	0.005**
Body image of caretaker	0.02	0.01	[−0.01, 0.04]	0.07	0.188
Self‐efficacy in education	−0.58	0.36	[−1.27, 0.12]	−0.09	0.107
Quality of life (proxy‐report of parent)	−0.01	0.02	[−0.04, 0.02]	−0.04	0.578
OB‐related quality of life (proxy‐report of parent)	−0.03	0.01	[−0.05, −0.01]	−0.22	0.001**
*R* ^*2*^ = 0.12, adj. *R* ^*2*^ = 0.10					

^a^ Significance codes: **p < 0.01; ***p < 0.001.

### 3.2 Determinants of RTC in Caretakers of Adolescents (Aged 12 and Older)

The mean level of RTC in the group of caretakers of adolescents was 6.13 (SD = 2.42). Table 4 shows the results of the correlation analyses. Only the variables OB‐related QoL (KINDL‐R OB module) and general QoL (KINDL‐R) reached significance and were therefore included in the multiple linear regression (Table 5). The model was able to significantly determine caretakers' RTC, *F*(2, 178) = 21.32, *p* < 0.001. The *R* ^2^ for the overall model was 0.19 (adjusted *R* ^2^ = 0.18). Only OB‐related QoL reached statistical significance (*β* = −0.44, *p* < 0.001).

### 3.3 Determinants of RTC in Caretakers of Children (Aged 11 and Younger)

The mean level of RTC in the group of caretakers of younger children was 6.03 (SD = 2.50). Table 4 shows the results of the correlation analyses between parental RTC and all possible determinants. The variables BMI‐SDS_LMS_ of children, self‐efficacy in education, body image of caretakers, QoL, and OB‐related QoL reached significance. Therefore, these variables were included in the multiple linear regression (Table 5). The model was able to statistically significantly determine caretakers' readiness, *F*(5, 309) = 8.04, *p* < 0.001. The *R* ^2^ was 0.12 (adjusted *R* ^2^ = 0.10). Of the five included variables, OB‐related QoL (*β* = −0.22, *p* = 0.001) and children's BMI‐SDS_LMS_ (*β* = 0.16, *p* = 0.005) could significantly determine parental readiness.

## 4 Discussion

The aim of the current study was to find variables that influence caretakers' and adolescents' motivation before starting a weight management program for pediatric OB and OW. Motivation was operationalized as RTC according to the TTM [21, 23]. In all samples, RTC was determined by OB‐related QoL of the children/adolescents. The RTC of caretakers of children was additionally determined by the children's BMI‐SDS_LMS_.

### 4.1 Determinants of Adolescents' Motivation

Adolescents' self‐reported RTC was influenced by their own OB‐related QoL. Furthermore, a trend to significance was identified for the relationship between RTC of adolescents' caretakers and their own. OB‐related QoL was negatively associated with the outcome, showing that a lower OB‐related QoL determines a higher RTC (interpreted as motivation to participate in the STARKIDS program). The relationship between caretakers' and adolescents' RTC was positive (although not quite reaching the level of significance), meaning that adolescents were more motivated in case their caretakers showed higher motivation, too.

The OB module of the QoL questionnaire (KINDL‐R) focuses on specific burden of children and adolescents with OB/OW, including somatic complaints (e.g., being out of breath), psychological burden (e.g., being sad because of high weight), and psychosocial difficulties (e.g., weight‐related bullying), and their impact on QoL. Data show that the higher the suffering triggered by OB or OW, the higher the motivation to end it. General QoL did not seem to have an influence on motivation, showing that adolescents are specifically suffering from complaints triggered by their weight. In addition, there was a clear trend to significance for adolescents' motivation being determined by their caretakers' or parents' motivation. Thus, caretakers' RTC might spill over to or was adopted by adolescents. This effect could help the whole family to begin and successfully continue a weight management program.

To the best of our knowledge, the study by Ramel et al. [24] was so far the only that assessed associations between different variables and children's own motivation. However, they reported children's data and no data of adolescents. Additionally, they did not include QoL or an OB‐related form thereof. Therefore, our study is the first to offer insight into factors that determine adolescents' motivation and could therefore be used to find similarities and differences between adolescents and their parents.

### 4.2 Determinants of Caretakers' Motivation

Results showed that RTC of caretakers of adolescents (aged 12 and older) is negatively associated with OB‐related QoL of the affected adolescents. Data used here were proxy estimates by the caretakers. However, results are similar for caretakers and their adolescents, as both are influenced by OB‐related QoL. In addition to adolescents, their caretakers might also perceive a high burden in the OB/OW, leading to a perception of lower QoL for their adolescents. This could increase their motivation to end their adolescents' suffering by participating in a weight management program.

RTC of caretakers of younger children was determined by the children's weight (BMI‐SDS_LMS_) and, again, caretakers' OB‐related QoL, but not general QoL. Like before, OB‐related QoL was negatively associated with motivation, showing that lower OB‐related QoL goes along with higher motivation, being measured as a proxy report of the caretakers. The relationship between the children's BMI‐SDS_LMS_ and the caretakers' motivation was positive, meaning that the higher the BMI‐SDS_LMS_ of the child (the more severe the OB of the child), the more the caretaker was motivated to participate in the STARKIDS program. This association seems reasonable, since a higher BMI‐SDS_LMS_ makes the OB more visible and impedes children, making it increasingly important to participate in weight management.

There is partial agreement between our results of caretaker data and existing evidence. Regarding parental weight, neither the finding of Maximova et al. [25] that less parental OW was associated with higher motivation nor the findings of Ramel et al. [24], who observed a positive relationship between parental BMI and RTC, could be found in our data since there was no association between parental BMI and parental motivation. However, as in Taylor et al. [26], we found a positive association between the children's weight status and parental motivation: caretakers were more motivated in case their children were more affected by OB. The age of the children in the study of Taylor et al. [26] was 4–8 years, which might explain why we also only observed this association between children's weight and caretakers' readiness in the sample of caretakers of the younger (11 years and younger) children, but not in the sample of caretakers of adolescents. As children grow older, this association seems to disappear. Another similarity between previous evidence [26] and our study is that parental motivation was dependent on the parents' concern about their children's weight [26] and on parental evaluation of their children's OB‐related QoL. Although these two variables (concern and OB‐related QoL) are not identical, they describe similar concepts, namely a worry or empathy about children or a perceived suffering.

### 4.3 Clinical Perspective

Data show that the determinants of RTC for both caretakers and adolescents were similar, as OB‐related QoL plays a major role in all models. Perspectives of caretakers and associated minors fit together showing that members of the same family share similar views. Additionally, all other significant variables were data from the respective counterparts: adolescents are influenced by their parents' motivation (clear trend to significance), parents (of younger children) are influenced by their children's weight, and parents are influenced by their minors' OB‐related QoL. In all groups nearly no associations between internal factors, such as own body image (in adolescents and caretakers), own BMI (in parents), or own self‐efficacy (in adolescents and caretakers), and RTC were observed. Almost all of the observed relationships were between two family members. We can interpret this as a unity of families where members share thoughts, problems, and resources and influence each other. This amplifies the usefulness to address the whole family in pediatric weight management, as it is also recommended in treatment guidelines [6, 7, 8]. Treatment of affected adolescents without inclusion of caretakers seems less useful in most cases which is why parents and caretakers are also called “agents of change” [56]. Fostering the whole families' motivation for change can be fruitful for improved and sustainable weight management. One helpful intervention therefore might be motivational interviewing with the whole family [57].

Transferring our findings into clinical practice, we need to have a closer look at OB‐related QoL. General QoL does not seem to have an influence on RTC. However, it is an important finding that specific OB‐related burden determines higher RTC for weight management in adolescents and caretakers. For future research, it is an important finding that families may be more motivated to participate in weight management programs once children and adolescents are already experiencing a lower OB‐related quality of life. Therefore, clinicians might rely on OB‐related QoL to increase motivation by providing education about the serious secondary diseases and psychosocial consequences resulting from OB and OW, but also by highlighting positive consequences of reducing OB and OW such as a better relationship to one's body or easier movement. In this context, the finding of Rhee et al. [58] is promising: explanations of the physician about strategies to reduce weight were related to parental readiness to change their children’s dietary behavior. Summarizing this, clinicians should work with the whole family, strengthen their collaboration, and should pay special attention to the OB‐related QoL of the affected children and adolescents.

### 4.4 Strengths and Limitations

To the best of our knowledge, this is the first study that investigated factors associated with adolescents' own motivation prior to a weight management program. Motivation was operationalized as RTC according to the widely used TTM [23]. Applying this common framework might lead to a better comparison of studies and results in the future [18]. The large sample size of the current analyses is another strength. Since data were assessed at baseline of a weight management program, all participants had already decided to take part in that program. Making the decision to take part in a weight management program reflects a certain level of motivation which might have led to a selection bias. In addition, the mean BMI percentile of participating children and adolescents is relatively high (98.46), indicating that the present data reflect the situation of severely affected families on the OB spectrum. Dropout has occurred before baseline data assessment. Since there are no data about these families, there is a lack of knowledge about a possible biased dropout. Furthermore, due to the cross‐sectional nature of the data analysis, it is not possible to interpret all observed determinants as demonstrating causality. Finally, RTC cannot be fully equated with a motivation to participate in the STARKIDS study but is more an approximation.

## 5 Conclusion

OB‐related QoL can determine caretakers' and adolescents' RTC before weight management. For clinicians it may be of interest that participation in family‐based weight management programs is dependent on the experience of a certain level of OB‐related burden. Additionally, our data suggest that the systemic family‐based focus of STARKIDS is highly relevant as motivational perspectives are reciprocally interdependent, at least between caretakers and adolescents.

## Author Contributions

Funding acquisition: Florian Junne, Guido Zurstiege, Inga Krauß, Isabelle Mack, Katrin Elisabeth Giel, Olaf Schliesing, Peter Martus, Stefan Ehehalt, Stephan Zipfel, Torben Sammet, and Wolfgang Bethge. Conceptualization of the STARKIDS intervention: Florian Junne, Guido Zurstiege, Inga Krauß, Isabelle Mack, Katrin Elisabeth Giel, Katrin Ziser, Stefan Ehehalt, and Stephan Zipfel. Conceptualization and methods of the STARKIDS study: Andreas Hoell, Florian Junne, Johann Jacoby, Katrin Elisabeth Giel, Katrin Ziser, Peter Martus, Stefan Ehehalt, Stephan Zipfel, and Wolfgang Bethge. Investigation in the STARKIDS study: Anne Herschbach, Alisa Weiland, Annalena Wels, Constanze Greule, Katrin Ziser, Rebecca Erschens, Maren Reyer, Monika Ums, Sophia Helen Adam, Olaf Schliesing, and Wolfgang Bethge. Project administration: Katrin Ziser and Rebecca Erschens. Conceptualization and methodology of the current study: Anne Herschbach, Katrin Elisabeth Giel, and Katrin Ziser. Formal analysis of the current study: Anne Herschbach. Supervision: Caterina Gawrilow, Florian Junne, Katrin Elisabeth Giel, and Katrin Ziser. Writing – original draft: Anne Herschbach. Writing – review and editing: all authors.

## Funding

The STARKIDS project is funded by the Innovation Committee of the German Joint Federal Committee (G‐BA) with the funding number 01NVF18013 (STARKIDS). The study funder has no role in the collection, management, analysis, and interpretation of the data. Part of this work has been supported by the European Union's Horizon Europe Research and Innovation Programme, grant agreement no. 101080219 (eprObes).

## Conflicts of Interest

The authors declare no conflicts of interest.

## Data Availability

The datasets analyzed during the current study and statistical code are available from the corresponding author on reasonable request.
